# HIV Vaccine: Recent Advances, Current Roadblocks, and Future Directions

**DOI:** 10.1155/2015/560347

**Published:** 2015-10-22

**Authors:** Muni Rubens, Venkataraghavan Ramamoorthy, Anshul Saxena, Nancy Shehadeh, Sandeep Appunni

**Affiliations:** ^1^Florida International University, 11200 SW 8th Street, AHC-4, No. 405, Miami, FL 33199, USA; ^2^Florida International University, 11200 SW 8th Street, AHC-5, No. 305, Miami, FL 33199, USA; ^3^Department of Biochemistry, All India Institute of Medical Sciences (AIIMS), New Delhi 110029, India

## Abstract

HIV/AIDS is a leading cause of mortality and morbidity worldwide. In spite of successful interventions and treatment protocols, an HIV vaccine would be the ultimate prevention and control strategy. Ever since identification of HIV/AIDS, there have been meticulous efforts for vaccine development. The specific aim of this paper is to review recent vaccine efficacy trials and associated advancements and discuss the current challenges and future directions. Recombinant DNA technologies greatly facilitated development of many viral products which were later incorporated into vectors for effective vaccines. Over the years, a number of scientific approaches have gained popularity and include the induction of neutralizing antibodies in late 1980s, induction of CD8 T cell in early 1990s, and combination approaches currently. Scientists have hypothesized that stimulation of right sequences of somatic hypermutations could induce broadly reactive neutralizing antibodies (bnAbs) capable of effective neutralization and viral elimination. Studies have shown that a number of host and viral factors affect these processes. Similarly, eliciting specific CD8 T cells immune responses through DNA vaccines hold future promises. In summary, future studies should focus on the continuous fight between host immune responses and ever-evasive viral factors for effective vaccines.

## 1. Introduction

Since the first recognized cases of the Acquired Immunodeficiency Syndrome (AIDS) came to light in the early 1980s and the discovery of the human immunodeficiency virus (HIV) soon after, HIV/AIDS has become a leading cause of mortality and morbidity worldwide. In the year 2013, global estimations showed that about 35 million people are living with HIV infection [1]. Since the initial identification and characterization of the disease, about 78 million people have become infected and 39 million people have died from AIDS related conditions [2]. However, the incidence of this disease has fallen by 38% since the year 2001 [3]. About 2.1 million people have become newly infected with HIV in the year 2013 compared to 3.4 million in the year 2001 [3]. AIDS related deaths have plummeted by 35% since the peak in the year 2005 [3]. In 2013, 1.5 million people died from AIDS related conditions compared to 2.4 million in the year 2005 [3]. Since the advent of antiretroviral medications, HIV infection has become a chronic disease with decreasing incidence and increasing prevalence.

In the year 2013, about 12.9 million people were receiving some form of antiretroviral therapy and constituted only 37% of all infected cases globally [4]. According to global estimates, about $19.1 billion was spent on HIV/AIDS and related conditions in the year 2013 and is estimated to increase to $24 billion by the year 2015 [5, 6]. This is a great burden on both developed and developing economies because more than 50% of total expenses are directed towards underdeveloped nations with decreased productive capacity and increased HIV associated life loss years. Though there are a number of effective prevention interventions and treatment methods like preexposure prophylaxis and antiretroviral therapy, researchers have always been zealous about HIV vaccine as the ultimate HIV prevention and control strategy. In spite of such efforts, there are only few studies that have shown successful results. The specific aim of this paper is to review recent vaccine efficacy trials and associated advancements about HIV vaccines and discuss the current challenges and future direction of this initiative.

## 2. Search Strategy and Selection Criteria

We followed a narrative review method to summarize recent advances in HIV vaccine development. We searched the electronic databases PubMed, EMBASE, Ovid, and Google Scholar for articles published between January 1985 and September 2015 (30 years) by combining the following search terms: “HIV”, “AIDS”, “vaccine”, “clinical trials”, “broadly neutralizing antibodies”, “CD8 T cells”, “CD4 T cells”, “antibody-dependent cell-mediated cytotoxicity”, and “antibody-dependent cell-mediated viral inhibition”.

## 3. Vaccine Efficacy Trials

Ever since HIV was formally identified as the cause of AIDS, there have been ongoing efforts on vaccines against the disease. On April 24, 1984, the US Secretary of Health and Human Services, Margaret Heckler, announced that vaccines will be researched and made ready for preliminary testing by the year 1986 [7]. However, this initial optimism was criticized by many eminent researchers because it failed to be coherent with existing knowledge about the pathophysiology and the mechanism of the virus itself. Traditional approaches of using live attenuated or whole inactivated viruses were considered unsafe because of the risk of permanently integrating proviral DNA within host chromosomes [8]. Advancements in vaccine development had to wait until mid-1980s when recombinant DNA technologies were becoming available for research applications. Following the success of recombinant Hepatitis B vaccine, recombinant DNA technologies were also being researched for HIV vaccines [9]. Rapid advances in the pathophysiology and molecular mechanisms of HIV enabled many structural components and proteins to be discovered and artificially synthesized through recombinant DNA technology. The culmination was the cloning and sequencing of HIV genome which led scientists to believe that an effective vaccine could be developed in the future. However, all these efforts came to a standstill with growing knowledge about extreme mutability and immune evasion mechanisms of existing HIV strains [10]. This was further complicated by the fact that neutralizing antibodies had no protective effects and their titers were similar among asymptomatic carriers and patients with active disease [11]. The exact mechanism of immunity against HIV is a puzzle and still remains unsolved. Currently 3 scientific paradigms have attracted researchers and include induction of neutralizing antibodies; induction of CD8 T cell mediated immunity; and combination approaches [12].

In spite of many nonmaterializing studies, hopes were renewed when a handful of studies presented newer approaches for inducing effective humoral and cell mediated immune responses against the virus. In a clinical trial done by Belshe et al. [13] in 1998, it was observed that live attenuated canarypox virus expressing HIV antigens were capable of inducing CD8 cytotoxic T cells against Env or Gag expressing target cells in 64% of the volunteers. It was also observed that the vaccine elicited profound neutralizing antibodies after vaccine priming and subunit boosting strategies. This study established the prime-boost concept for future HIV vaccine research. This study also showed that such prime-boost regimens induced both cellular and humoral responses. However, in another large scale clinical trial done among 330 healthy volunteers, canarypox vaccine (vCP1452) failed to induce adequate CD8 cytotoxic T lymphocyte responses or neutralizing antibodies measured by enzyme-linked immunoassay (ELISA) thereby dampening the hopes generated in previous studies [14].

In a study (RV144) done among 16,402 healthy participants to test the efficacy of recombinant canarypox vector vaccine (priming by four doses of ALVAC-HIV [vCP1521] and booster by two doses of AIDSVAX B/E), a vaccine efficacy of 31.2% (95% CI, 1.1 to 52.1) was observed [15]. The study also suggested that antibodies (IgG_1_ and IgG_3_) directed towards V1/V2 region of gp120 showed some protective effects against transmission of HIV-1 infection. In addition, IgA antibodies towards envelope proteins were inversely associated with mucosal HIV-1 transmission. However, vaccination did not affect the immunological (CD4 cell count) and virological markers (HIV viral RNA) in subsequently diagnosed HIV infected participants. In spite of modest results shown by RV144 trial, the reasons for the decreased risk of infections in vaccinated subjects with antibodies against V1/V2 region of gp120 are a remarkable finding. Thus, V1/V2 constitutes an important component in the process of viral integration into the host cell. V1/V2 region of gp120 interacts with CD4 receptors as well as gut mucosal homing integrin binding site *α*4*β*7 of CC chemokine receptor 5 (CCR5) coreceptor, resulting in incorporation of the viral genome into the host cell [16, 17].

As a follow-up of RV144 study, the HVTN P5 was proposed to research a pox-protein based HIV vaccine. The potential candidates selected were ALVAC-C (expressing ZM96 gp120 (clade C strain) linked to gp41, and gag and pro clade B LAI strain); NYVAC-C (bivalent highly attenuated vaccinia virus expressing clade C ZM96 gp140 and ZM96 Gag-CN54 Pol-Nef fusion proteins); Gp120 protein + MF59 (clade C TV1 gp120 Env and clade C 1086 gp120 Env with MF59 adjuvant); Gp120 protein + ASO1B (clade C TV1 gp120 Env and clade C 1086 gp120 Env with ASO1B adjuvant); and DNA-C (trivalent DNA expressing clade C ZM96 Gag and gp140, and a CN54 Pol-Nef fusion construct). This multisite international study selected sites in Southern Africa and Thailand. This study has prospects for next generation clade C-adapted vaccines with more effective priming immunogens and adjuvants with potent immunological properties.

In another large scale multisite study (HVTN 505) done among 2,504 participants, a 6-plasmid DNA vaccine with embedded gag/pol/env/nef proteins from clades A, B, and C was administered at 0, 4, and 8 weeks [18]. This was followed by booster vaccine, rAd5, expressing clade B gag-pol fusion protein and env glycoproteins from clades A, B, and C. Results showed that the vaccine was not efficient in spite of its acceptable side effect profile and the study was discontinued for the same reasons. This study also revealed that DNA/rAd5 vaccine regimen did not produce any changes in the rate of HIV transmission or plasma viral load.  Table 1 shows the list of all HIV vaccine efficacy trials.

## 4. Broadly Reactive Neutralizing Antibodies (bnAbs)

Protective immune response in HIV infection is an ultimate challenge because of the characteristics of the virus itself. HIV virus mutates very rapidly leading to many changes in the envelope proteins within a short time span. A vaccine therefore should elicit a number of antibodies capable of neutralizing many genetically different strains. Majority of HIV infected patients show a prompt monoclonal type antibody response capable of affording some levels of protection against the virus. However, the virus develops resistance to these antibodies and thrives in the host superseding the humoral and cellular immune responses. Notable exceptions to this are nonconventional bnAbs which are observed in a very small percentage of HIV infected individuals [19]. Even in those individuals who produce bnAbs, only about a quarter are capable of inducing cross-reacting antibodies with adequate breadth and potency measured by standardized neutralization assays [20]. Such bnAbs are known to develop during the first three years of natural infection. Current researchers opine that vaccine regimens should focus on inducing useful bnAbs for neutralization of the viral strains thereby providing high levels of protection. Though this is very promising, bnAbs also have shortcomings. BnAbs are very rarely produced and the mechanisms for inducing them through feasible vaccination regimes are not yet fully understood. BnAbs undergo extensive somatic hypermutation thereby developing extreme specificity for viral strains as well as increasing the breadth and potency of HIV viral neutralizations [21]. However, it is difficult to induce bnAbs production because of several levels of somatic hypermutations needed for the process which takes months to years, by which time the virus develops newer and resistant mutations [21, 22]. Researchers have also expressed concerns about bnAbs being polyreactive or autoreactive and potentially harmful, and therefore balancing the beneficial versus adverse effects needs further consideration [23]. Such lineages of B cells and plasma cells producing autoreactive antibodies are also eliminated through natural apoptotic mechanisms thus terminating the production and maturation of bnAbs.

Currently, many neutralizing antibody targets are being researched as potential candidates for inducing bnAbs production. These include receptor binding sites on gp120 for CD4 receptors and CCR5 or CXCR4 coreceptors; variable regions like V1, V2, and V3 on gp120; and binding sites on gp41 molecules such as conserved helices and membrane-proximal external region (MPER) of gp41 [24]. In a study done among rhesus macaques, it was observed that passively administered b12 bnAb provided adequate protection against huge doses of simian-human immunodeficiency virus (SHIV) inoculated into these animals [25]. This study postulates that affordable levels of neutralization and protection against HIV can be achieved in humans through passive administration of bnAbs. This would be more effective in humans because, compared to experimental animals, humans experience much lower levels of viral transmission through unprotected sex and other means of transmission. This study provides hope for passive immunization schedules because a Cox proportional hazard ratio of 21.3 (95% CI: 1.7–260.9) was observed for b12 bnAb compared to control antibodies thereby affording 21 times more protection from SHIV challenge [25]. However, SHIV and HIV cannot be entirely compared because of differences in receptors involved in viral integration. Similarly, a sequel to this study by the same researcher showed that another bnAb, 2G12, offered stronger protection than b12 with serum neutralizing titers as high as 1 : 1, compared to approximately 1 : 100 for b12 bnAb [26]. This has been ascribed to immediate antibody-virus neutralization by 2G12, compared to much slower processes by b12 bnAb. This study stresses the importance of 2G12 as very effective prevention strategy since it provides immediate protection against target cell infection and integration of the virus. However, other studies refute these claims and demonstrate that both b12 and 2G12 did not achieve the expected level of neutralization. In a study by Hraber et al., [27] it was observed that b12 and 2G12 in 50 *μ*g/mL concentrations neutralized only 29.4% and 20.2% of total number of strains of viruses tested in the study.

Another study was done among rhesus macaques in order to estimate the effects of passive administration of bnAbs. This study suggested that the administration of 5 mg/kg of PGT121 antibody produced complete neutralization of SHIV_SF162P3_ strains and similar effects were found with 1 mg/kg doses [28]. However, with 0.2 mg/kg of PGT121 and placebo antibodies the macaques started to get infected with SHIV_SF162P3_. This study concludes that bnAbs provide total protection against viral transmission when administered passively in sufficient doses. A number of animal studies have shown that bnAbs produce therapeutic neutralization of viruses in infected animals. In the long run, bnAbs could evolve into a complimentary therapeutic modality in addition to antiretroviral medications and preventive approaches. In a study by Klein et al. [29], the effects of 5 bnAbs were tested in mice with HIV-1_YU2_-infection. The antibodies tested included 45-46^G54W^, PG16, PGT128, 10-1074, and 3BC176. The study showed a net decrease of 1.1 log_10_ for PGT128, 1.5 log_10_ for 10-1074, 0.23 log_10_ for PG16, 0.56 log_10_ for 45-46^G54W^, and no effects for 3BC176. However, the viremia returned to untreated levels within 14–16 days after the administration of these passive antibodies in all except one mice. Thus, bnAbs produced only transient decrease in viral load in humanized mice with HIV-1_YU2_-infection. The study concludes that, in the future, advancements and combinations of such bnAbs could produce effective long-term control of HIV-1 viremia and could be used for human beings as well. In a study by Barouch et al. [30], PGT121, 3BNC117, and b12 were administered in doses of 10 mg/kg to SHIV_SF162P3_ infected rhesus monkeys. There was significant and long-term control of viremia in animals with low baseline viral loads (<3.5 log RNA copies/mL), intermediate but short-term control of viremia in animals with intermediate viral loads (3.5–5.3 log RNA copies/mL), and incomplete control and rapid rebound viremia in animals with high baseline viral loads (>5.3 log RNA copies/mL). The study shows that bnAbs though effective for HIV treatments have ceiling effects and cannot be used as a sole modality for treatment and need to be reinforced with antiretroviral medications. In another study by Shingai et al. [31], 3BNC117 and 10-1074 blocked infection and suppressed viremia in macaques infected with R5 tropic SHIV-AD8. However, both the therapies resulted in rebound viremia by day 10 and day 20, respectively, of treatment initiations. Single genome amplification (SGA) studies later confirmed that the virus mutated and lost the gp120 Asn 332 glycan in both of the antibody treated animals rendering the virus resistant to both the antibody treatments.  Table 2 shows broadly neutralizing antibodies, target sites, and their breadth of neutralization.

## 5. Coevolution of Broadly Neutralizing Antibodies

The neutralizing antibodies in the HIV infection should be able to cope with a number of immune evasion strategies acquired by the viruses. The majority of the neutralizing antibodies are directed towards the Env, which the viruses keep mutating to evade the host immune responses [41]. To overcome this phenomenon, successful vaccines should induce antibodies that are capable of binding and neutralizing a broad spectrum of circulating viral products [42]. Such bnAbs are found only in a small percentage of natural infection and a set of host and viral factors mediate the development as well as the breadth and potency of bnAbs. Host responses include the genetic propensity towards stimulating and utilizing VH gene, which are typically found in the allele IGHV1-2^*∗*^02. Allelic differences in activation of VH gene explain the existence of different branches of somatic hypermutations from common germline sequences. In HIV infected individuals with IGHV1-2^*∗*^02 alleles, the somatic hypermutation is geared towards production of useful bnAbs [33, 35]. Future vaccine trials should focus on stimulating VH genes for effective bnAbs production. Other important host factors include the number of circulating and functional CD4 T cells in peripheral blood as well as germinal centers where the affinity maturation occurs. CD4 T follicular helper cells are necessary for B cell activation and production of germinal centers [43]. In a study among HIV positive participants, a specialized subpopulation of circulating memory PD-1^+^CXCR5^+^ CD4 T cells in peripheral blood was also positively associated with bnAbs production [44]. Several viral factors are also associated with production of bnAbs. BnAbs are generally produced in patients with moderate and sustained viral load [42, 45]. Furthermore, viruses should undergo a specific number of mutations of Env epitope targets after the development of precursors of bnAbs. Only then would bnAbs mature through a specific sequence of somatic hypermutations and retain the germ line sequences and affinity for target sites. Thus it is a complex interplay of host antibody acquisition potentials and ever-evading viral mutations that generate and sustain useful neutralizing bnAbs. A few studies have tried to explain the evolution of bnAbs responses in HIV positive patients as well as understand the mechanisms by which bnAbs are produced. In a study done by Liao et al. [22], it was found that bnAb CH103 neutralized approximately 55% of HIV-1 products. This study also found that a cocrystal bound structure of CH103 bnAbs and gp120 produced increased affinity for CD4 binding site and the right sequence of somatic hypermutation for production of bnAbs. This study further showed that extensive viral diversification of CH103 epitope facilitated increased neutralization breadth of existing antibodies. CH103 epitope constitutes an important target for vaccination schedules to induce useful neutralizing bnAbs. A subsequent study by Fera et al. [46] further explains the evolution of bnAbs in HIV infected individuals. According to this study, bnAb CH103 interacted with gp120 through heavy-chain complementarity determining region 3 (CDRH3) to produce conformational changes in the orientations of heavy and light chains and thereby produced increased viral neutralization response. This study opines that channelizing the continuous struggle between virus and host immune responses could help scientists to develop effective vaccines that stimulate useful bnAb production.

## 6. Antibody-Dependent Cell-Mediated Cytotoxicity (ADCC) and Antibody-Dependent Cell-Mediated Viral Inhibition (ADCVI)

Controlled viral load and better immunological profiles were achieved through potent and persistent ADCC and ADCVI [47, 48]. Both ADCC and ADVI are initiated by HIV-1 binding antibodies attaching to the Fc receptors of natural killer (NK) cells, enabling them to destroy infected cells expressing the HIV viral antigens [49]. The strength of ADCC is measured by the ability of NK cells to destroy HIV infected cells. Similarly, ADCVI is measured by the ability of virus-specific antibodies and NK cells to control or inhibit HIV viral replication in a population of infected cells. Both ADCC and ADCVI are mediated by antibodies that develop early in the course of infection and have a broad neutralizing spectrum enabling NK cells to have better reactivity profiles in the initial stages of HIV infection [50]. However, the viral replication rate and associated mutations outpace the rates of ADCC and ADCVI activity thereby establishing the infection in the target cells. Majority of the studies focusing on HIV vaccines are directed towards accelerating the pace of ADCC and ADCVI before the virus gets mutated thereby increasing the elimination of HIV before it establishes in the host cells [51]. In a study by Asmal et al. [52] on SIV infected rhesus macaques, effective ADCVI inhibited SIV replication by 100 times. This was observed during the first 3 weeks of SIV infection before the development of ineffective plasma antibodies having low affinity for the mutated virus. Many of the mutated SIV envelope glycoproteins during late stages of the infection were susceptible to ADCVI activity though they were immune to plasma neutralization. This study showed that SIV with mutated envelope glycoproteins could not escape ADCC and ADCVI [52]. In a human study, antibodies responsible for ADCC and ADCVI provided prolonged protections from HIV-1 strains thereby controlling the infection and affording long-term immunity [53]. In another study, it was found that increased ADCC and ADCVI activity had reduced the risk of vertical transmission in women with high viral loads. This study also showed that these antibodies were transferred to the infants through breast milk [54].

## 7. Stimulation of CD8 T Cells Immune Responses

Another broad arm of vaccine development studies includes eliciting HIV specific CD8 T cell responses. CD8 T cells destroy viruses and infected cells thereby helping in the process of convalescence in any viral infection. This mechanism does not work with HIV because CD8 T cells control the viremia but cannot eliminate it [55]. In a study done by Barouch et al. [56], it was found that DNA vaccines elicited potent CD8 T cell responses and stable CD4 T cell counts which were improved by purified fusion protein IL-2/Ig. However, the STEP trial which administered MRKAd5 HIV-1 gag/pol/nef trivalent vaccine failed to show similar results and rather increased the risk for HIV infection [57]. Similarly, in the HVTN 505 trial, stimulation of CD8 T cells failed to show any protective effects on HIV infection [18]. Failure of these trials challenged the very concept of CD8 T cells immune responses for prevention of HIV. In spite of such disappointing results, recent studies have focused some viral vectors for stimulating and sustaining useful CD8 T cell activity. Vaccination with SIV protein-expressing rhesus cytomegalovirus (RhCMV/SIV) vectors offered long-term control over viral load through stimulation of CD8 T cells immune responses. For example, in a study done by Hansen et al. [58], it was observed that virus specific effector memory T cells maintained through effective vectors completely stopped the replication of SIV infection and also maintained notable over time immune surveillance. This study suggests that vaccines targeting effector memory T cells could possibly afford functional cure and eradication of HIV infection as observed in SIV infected rhesus macaques. Thus, we see that CMV vectors induce uninterrupted, powerful, and long-term antiviral immune surveillance over SIV infection and they are promising candidate for vaccine development studies not only for HIV but also for other viral diseases.

Recent advances in T cell based vaccines have focused on incorporating the near complete gene sequences of several proteins expressed by the viral strains in HIV controllers. These composite immunogens aim at maximizing the incorporation of variable viral epitopes or* mosaics* [59]. Another vaccine strategy includes avoiding all variable viral epitopes and incorporating only* conserved regions* [60]. In a study among rhesus monkeys, it was observed that the mosaic antigens incorporating several phenotypes of HIV-1 Gag, Pol, and Env antigens administered through replication-incompetent adenovirus serotype 26 vectors markedly increased the depth and breadth of T lymphocyte responses [59]. In a humans study, vaccines incorporating the conserved regions of HIV-1 proteome induced T cells which recognized infected CD4 cells and decreased HIV-1 viral replication [60]. Both the studies independently show the importance of either construct for successful vaccine development strategies through advanced targeting of HIV viral proteome.

## 8. Viral Vectors, Alternative Delivery Systems, and Costimulatory Molecules

A number of viral vectors and alternative delivery systems are being researched for advanced HIV vaccines. These include vectors such as nonreplicating adenovirus (Ad), adeno-associated virus (AAV), Venezuelan equine encephalitis virus (VEE), Sindbis virus (SIN), herpes simplex virus (HSV), Measles virus (MV), modified vaccinia virus Ankara (MVA), vesicular stomatitis virus (VSV), canarypox (ALVAC), Semliki forest virus (SFV), DNA vectors, mRNA vectors, and nanoformulations [61].  Table 3 shows examples of viral vectors and alternative HIV vaccine delivery systems.

Costimulatory molecules provide secondary signals for additional activation of T cells for increasing vaccine mediated immune response. For example, costimulatory molecule B7.2 delivered through the vector HIV pCgag/pol enhanced the functioning of cytotoxic T lymphocytes [62]. Similarly, TNF superfamily ligands (TNFSFL) have been shown to improve the efficacy of ALVAC HIV-1 vaccines in a Phase III clinical trial [63]. Several other costimulatory molecules are being researched for increasing the efficacy of newer vaccines.

## 9. Future Challenges and Directions

In summary, we can say that there are several unanswered questions from many failed as well as marginally successful studies. A number of studies have identified potential epitopes for bnAbs which include V1/V2, V3 glycan, and CD4 binding site. The most important challenge is which of these epitopes need to be targeted by future vaccines. The further question is whether vaccines should focus on individual epitopes or a combination of multiple epitopes. Researchers should also focus on the breadth, magnitude, and durability and other characteristics that make bnAbs significantly neutralizing and useful. Future studies should also focus on elucidating the right sequence of somatic hypermutations to derive effectively neutralizing bnAbs. The phenomenon of inducing somatic hypermutation through vaccination itself is not fully understood and requires further research. Even in studies showing marginal effects, the long-term efficacy of vaccines is questionable. This factor is very important because HIV/AIDS is becoming a chronic disease with increasing prevalence, long number of infected years, and associated risk for transmission. Vaccines therefore need to be effective in the long term and offer continuous protection. With regard to stimulation of cytotoxic T cells, the exact mechanism for the production of effective CD8 T cell responses needs to be researched and understood. Furthermore, results obtained from studies on rhesus macaques with SIV viral proteins embedded on CMV vectors cannot be entirely expected to produce analogous results in HIV viral proteins embedded vector vaccines in human beings due to the phylogenetic differences between simian and human immunodeficiency viruses. Finally, the very emergence and continuous evolution of innumerable number of quasi-species of HIV with diversities in genetic sequences and expressed surface glycoproteins pose great challenges for HIV vaccines.

## 10. Conclusion

Many new immunological and virological markers like proviral DNA levels in reservoir cells, measures of functional T and B cell subsets, markers of biochemical and cellular immune response, viral transmission rates, and viral neutralization rates should be used in future studies to evaluate efficacy outcomes in large-scale studies. This is especially challenging for existing researchers and funding institutions because newer paradigms are being proposed and discarded rapidly and research on these paradigms is expensive and complicated. The more time and money we invested in large-scale studies, the closer we are to a successful vaccine. The governments of different nations should cooperate together in meeting these investments for a cause that could ultimately save millions of lives as well as the money spent on managing and treating HIV/AIDS. Only then can HIV/AIDS be controlled even in the poorest nations across the globe and hopes of eradication in the future would become a reality.

## Figures and Tables

**Table 1:** HIV vaccine efficacy trials.

Study	Site	Vaccine	Volunteers	Vaccine to placebo randomization	Results
Vax004	USA and Netherlands	AIDSVAX B/B′ gp120 with alum	5,100 MSM and 300 women	2 : 1	No vaccine efficacy

Vax003	Thailand	AIDSVAX B/E gp120 with alum	2,500 men and women IDUs	1 : 1	No vaccine efficacy

HVTN 502 Step trial	North America, the Caribbean, South America, and Australia	MRKAd5 HIV-1 gag/pol/nef trivalent vaccine based on adenovirus type 5	3,000 MSM and heterosexual women and men	1 : 1	No vaccine efficacy

RV144	Thailand	Recombinant canarypox vector vaccine (ALVAC-HIV [vCP1521]) and recombinant glycoprotein 120 subunit vaccine (AIDSVAX B/E)	16,402 community-risk men and women	1 : 1	31.2% vaccine efficacy at 42 months

HVTN 503 Phambili trial	South Africa	MRKAd5 HIV-1 gag/pol/nef trivalent vaccine based on adenovirus type 5	801 heterosexual men and women	1 : 1	No vaccine efficacy

HVTN 505	USA	6-plasmid DNA vaccine and rAd5 vector boost	2,504 men or transgender women who have sex with men	1 : 1	No vaccine efficacy

HIV-V-A004	USA, Rwanda, South Africa, Thailand, and Uganda	Homologous Ad26 mosaic vector regimens or Ad26 mosaic and MVA mosaic heterologous vector regimens, with high-dose, low-dose or no clade C gp140 protein plus adjuvant	400 men and women	—	Results awaited

HVTN 100	South Africa	Clade C ALVAC-HIV (vCP2438) and bivalent subtype C gp120/MF59	252 men and women	5 : 1	Results awaited

HVTN 702	South Africa	ALVAC-HIV and bivalent subtype C gp120/MF59	5,400 men and women	1 : 1	Results awaited

HVTN 703/HPTN 081	South America and Sub-Saharan Africa	VRC01 broadly neutralizing monoclonal antibody	2400 MSM and transgender and 1500 women	2 : 1	Results awaited

Note: MSM: men who have sex with men; IDUs: IV drug users.

**Table 2:** Broadly neutralizing antibodies, target sites, and breadth of neutralization.

Broadly neutralizing antibodies	Target site	Breadth of neutralization (IC_50_ < 50 *μ*g/mL)	Study
VRC01	CD4 binding site	89% of 180 isolates	Huang et al. (2012) [32]
VRC02	CD4 binding site	91% of 190 isolates	Wu et al. (2010) [33]
VRC03	CD4 binding site	57% of 190 isolates	Wu et al. (2010) [33]
VRC-PGV04	CD4 binding site	88% of 162 isolates	Walker et al. (2011) [34]
VRC-PGV04b	CD4 binding site	71% of 178 isolates	Wu et al. (2011) [35]
CH103	CD4 binding site	55% of 196 isolates	Liao et al. (2013) [22]
2F5	gp41	39% of 92 isolates	Corti et al. (2010) [36]
2G12	gp120	41% of 90 isolates	Binley et al. (2004) [37]
4E10	gp41	98% of 162 isolates	Walker et al. (2009) [38]
PG9	V1-V2 loops	78% of 180 isolates	Huang et al. (2012) [32]
PGT130	V3 loop	52% of 162 isolates	Walker et al. (2011) [34]
PGT151	gp120-gp41	66% of 117 cross-clade isolates	Bonsignori et al. (2014) [39]
PGT152	gp120-gp41	64% of 117 cross-clade isolates	Blattner et al. (2014) [40]

Source: http://www.hiv.lanl.gov/.

**Table 3:** Examples of viral vectors and alternative HIV vaccine delivery systems.

Examples of vectors	Examples of vaccine
Nonreplicating adenovirus vectors (Ad)	Mixture of 4 rAd5 vectors that express HIV-1 subtype B Gag-Pol fusion protein and envelope (Env) from subtypes A, B, and C

Adeno-associated virus (AAV)	Adeno-associated virus based HIV-1 subtype C vaccine (tgAAC09)

Venezuelan equine encephalitis virus (VEE) Sindbis virus (SIN)	Recombinant trimeric HIVΔV2gp140Env protein

Herpes virus (HSV)	Recombinant herpes simplex virus (HSV) envelope and Nef antigens of simian immunodeficiency virus

Measles virus (MV)	Recombinant measles virus vaccines expressing HIV-1 clade B envelope glycoprotein

Modified vaccinia virus Ankara (MVA)	Modified vaccinia virus Ankara-vectored HIV-1 clade A vaccine

Vesicular stomatitis virus (VSV)	Recombinant vesicular stomatitis virus- (rVSV-) based vectors expressing HIV-1 env 89.6P gp160

Canarypox (ALVAC)	HIV-1 canarypox vaccine (vCP1452)

Semliki forest virus (SFV)	Self-amplifying rSFV2gen RNA encoding HIV-1C antigens

DNA vectors	HIV-1 env/rev DNA vaccine

mRNA vectors	MS2 VLP-mediated RNA vaccine

Nanoformulations	Fullerenol: nanoformulation of virus sized nanoparticles with dual-function nanoadjuvants to simulate immune responses to the HIV DNA vaccine
